# Local Respiratory Allergy: From Rhinitis Phenotype to Disease Spectrum

**DOI:** 10.3389/fimmu.2021.691964

**Published:** 2021-06-02

**Authors:** Almudena Testera-Montes, Maria Salas, Francisca Palomares, Adriana Ariza, María J. Torres, Carmen Rondón, Ibon Eguiluz-Gracia

**Affiliations:** ^1^ Allergy Unit, Hospital Regional Universitario de Malaga, Malaga, Spain; ^2^ Allergy Group, Instituto de Investigación Biomédica de Málaga-IBIMA and Red Tematica de Investigacion Colaborativa en Salud (RETICS) de Asma, Reacciones Adversas y Alergicas (ARADyAL), Málaga, Spain; ^3^ Department of Medicine and Dermatology, Universidad de Malaga, Malaga, Spain; ^4^ Laboratory for Nanostructures for the Diagnosis and Treatment of Allergic Diseases, Andalusian Center for Nanomedicine and Biotechnology (BIONAND), Malaga, Spain

**Keywords:** allergic rhinitis, dual allergic rhinitis, local allergic rhinitis, local allergic asthma, local respiratory allergy, mucosal immunology, IgE synthesis

## Abstract

Local respiratory allergy (LRA) is defined by the negativity of atopy tests, a clinical history suggestive of airway allergy and a positive response to the nasal and/or bronchial allergen challenge. The clinical spectrum of LRA is comprised of three conditions: local allergic rhinitis (LAR) and local allergic asthma in non-atopic patients, and dual allergic rhinitis (coexistence of allergic rhinitis and LAR) in atopic individuals. LRA is an independent disease phenotype not progressing to atopy over time, but naturally evolving to the clinical worsening and the onset of comorbidities. Published data suggests that LRA is mediated through the mucosal synthesis of allergen-specific (s)IgE, which binds to FcϵRI on resident mast cells, and in >50% of cases traffics to the blood stream to sensitize circulating basophils. To date, 4 clinical trials have demonstrated the capacity of allergen immunotherapy (AIT) to decrease nasal, conjunctival and bronchial symptoms, to improve quality of life, to increase the threshold dose of allergen eliciting respiratory symptoms, and to induce serum sIgG_4_ in LRA individuals. Collectively, these data indicate that local allergy is a relevant disease mechanisms in both atopic and non-atopic patients with airway diseases.

## Introduction

Allergic rhinitis (AR) and allergic asthma (AA) are very common diseases (1, 2) and account for high direct and indirect costs (3). These conditions have been historically identified by the positivity of atopy tests (skin-prick test (SPT) and/or serum allergen-specific (s)IgE) (4, 5). However, these tests are only indicative of sensitization (6), and the confirmation of the allergic etiology of airway diseases sometimes requires a nasal or bronchial allergen challenge (NAC and BAC, respectively) (7). Of note, the atopy-based classification of airway diseases is being gradually replaced by an allergy-grounded division based on the presence of airway allergen-specific reactivity. In this regard, AR and AA patients display positive NAC or BAC results, together with positive SPT and detectable serum sIgE (8). Importantly, some non-atopic subjects with rhinitis and asthma also display positive NAC and BAC responses, and these phenotypes have been termed local allergic rhinitis (LAR) (9) and local allergic asthma (LAA) (10), respectively. Moreover, recent data indicate that AR and LAR can occur in the same rhinitis patient, and this scenario has been called dual allergic rhinitis (DAR) (11). Collectively, these observations suggest that local allergic mechanisms drive a broad spectrum of airway diseases (local respiratory allergy, LRA) in both atopic and non-atopic patients. In this review we will summarize the main clinical and immunological features of LAR, DAR and LAA individuals.

## Clinical Phenotypes of Local Respiratory Allergy

LAR was the first condition identified within LRA spectrum (9), and thus it is also the best studied one. The disease is characterized by chronic nasal symptoms suggestive of allergy in a patient testing negative for SPT and serum sIgE, but who displays a positive NAC (12, 13). As compared to non-allergic rhinitis (NAR) subjects, LAR patients are more frequently young women who do not smoke but who have a family history of atopy, more severe nasal symptoms and an earlier rhinitis onset (12). LAR is a differentiated rhinitis phenotype, not progressing to atopy over time, but naturally evolving towards the aggravation and the association of conjunctivitis and asthma (14). In this regard, the prevalence of asthmatic symptoms in LAR patients, increased significantly from 19% to 31% during the 10 years following rhinitis onset (14). This finding prompted us to characterize the bronchial symptoms in LAR patients. By conducting methacholine provocations, we confirmed asthma diagnosis in 50% of LAR patients reporting asthma-guide symptoms (10). Moreover 29% of these patients displayed also positive BAC results, thus demonstrating the existence of a bronchial counterpart of LRA (LAA). Importantly, the BAC induced a sustained increase of airway hyper-responsiveness in LAA patients (10). Of note, a recent study conducted in Poland, confirmed the presence of asthma and LAA in 76% and 58% of LAR patients reporting asthmatic symptoms, respectively (15). Both pollens (e.g. grass and birch pollens), house dust mites (HDM) and *Alternaria alternata* can trigger LRA symptoms (13).

The implementation of NAC and BAC in the clinic allows for a more detailed phenotyping of rhinitis and asthma patients (16). In the clinical practice, the results of atopy tests do not always match the patient’s pattern of nasal symptom (e.g. perennial rhinitis with positive SPT to seasonal pollens only) (6). This scenario was classically explained by the coexistence of allergic and non-allergic rhinitis in the same patient, a phenomenon defining the mixed rhinitis (MR) phenotype (17). Nevertheless, our group identified that 85% of adult patients sensitized to seasonal pollens only, but suffering from perennial rhinitis, tested positive to the NAC with perennial allergens and with seasonal allergens (11). This clinical scenario was called DAR, and a later study demonstrated its relevance also in pediatric populations (18). Compared to MR patients, nasal symptoms in DAR subjects start more often with a seasonal pattern with subsequent evolution to perennial rhinitis. Conversely, nasal hyper-reactivity is more common among MR individuals (11).

## Local respiratory allergy across the lifespan

No population study has investigated yet the global prevalence of LRA. A 10-year follow-up study showed that LAR onset occurs during childhood in 36% of cases (14). In this regard, a systematic review concluded that 16% of non-atopic children with rhinitis fulfill diagnostic criteria for LAR (19). Nevertheless, regional differences in LAR prevalence might exist, as published data consistently identify a higher frequency of LAR among Western (29-67%) (20–23) as compared to Asian (0-19%) (24–27) non-atopic children with rhinitis. To address this aspects, we recently conducted a systematic evaluation (SPT, serum sIgE and NAC) of pediatric patients referred to specialized units for chronic nasal symptoms, and found a 46%, 12%, 25% and 18% prevalence for AR, DAR, LAR and NAR, respectively (18). The clinical profile was comparable among patients with the allergic phenotypes of rhinitis, but allergic subjects had an earlier disease onset, higher prevalence of conjunctivitis, and more severe nasal symptoms than NAR patients. Overall, these data indicate that LRA is totally or partially involved in 36.5% of cases of pediatric rhinitis. On the other hand, LAA phenotype has not been described yet in children or adolescents.

Studies focusing on adult populations report an 8-70% prevalence of LAR among non-atopic individuals with rhinitis, even though most works communicate frequencies over 50% and the trend towards a lower prevalence in Asian countries is less pronounced (24, 28–31). Importantly, the above mentioned systematic review concluded that the global possibility for a positive NAC in adult non-atopic patients with rhinitis was 25% (19). Conversely, there is no reliable data about DAR prevalence in adults, as the only study investigating the phenotype in patients >18 years, included highly selected individuals who are unlikely representative of the general population (11). Similarly, LAA phenotype has been only investigated in adult LAR patients reporting asthma-guide symptoms and displaying FEV1>80% (10, 15). Of note, all study individuals testing positive to the methacholine provocation suffered from mild-to-moderate asthma. Thus, the reported prevalence (29% and 58% in the Spanish and Polish studies, respectively) can hardly reflect the real frequency of LAA. The only study investigating LAR relevance in elderly patients reported a 21% prevalence of the disease among rhinitis subjects >65 years (32). DAR and LAA phenotypes have not been described yet among elderly populations.

Collectively, these data indicate that LRA is a common entity across the entire lifespan.

## Immunopathology of Local Respiratory Allergy

Airway allergen-specific reactivity is associated with eosinophilic inflammation, regardless of the patient’s atopic status for the corresponding allergen. In AR, DAR, MR and LAR patients, eosinophil cationic protein (ECP) increases during the 24 hours after a positive NAC (11, 33, 34). Similarly, eosinophils and ECP increase in the sputum of AA and LAA patients 24 hours after a positive BAC (10). Conversely, a negative NAC or BAC does not modify nasal or sputum ECP, even in atopic patients (10, 11, 33). Moreover, a positive NAC also induces an early and short increase of nasal tryptase in LAR individuals (33, 34). Collectively, these observations indicate that LRA is associated with the classical early and late reactions of allergic inflammation.

The comprehension of the role of sIgE in LRA, requires understanding the immunobiology of this antibody isotype (35). Mouse models and clinical studies of AR and AA indicate that CD1c+ myeloid dendritic cells excel in priming allergen-specific Th2 lymphocytes during the sensitization phase of allergic inflammation (36, 37). Activated Th2 cells communicate with naïve B cells in the secondary lymphoid tissues to trigger their class switch recombination to IgE (ϵCSR) (35). However, IgE-switched B cells are unable to perform efficient somatic hypermutation in the germinal centers (38). Therefore, germinal center-derived sIgE is very rare and displays and insufficient affinity maturation. On the other hand, IgG_1_-switched B cells can acquire a mature phenotype in the secondary lymphoid tissues and gain access to systemic circulation (38). When AR or AA patients are re-exposed to the allergen, vast numbers of monocytes are recruited to the airway mucosa, where they differentiate into inflammatory dendritic cells to locally reactivate memory Th2 cells (36, 39, 40). Importantly, LAA patients also experience an increase of sputum monocytes 24 hours after a positive BAC (10). Reactivated memory Th2 cells release IL-4 which mediates the sequential ϵCSR of IgG_1_+ B cells in the airway mucosa (41). Ample evidence indicate that most sIgE in AR patients (42–46), and to a lesser extent in AA subjects (47, 48), is synthesized at the mucosal level through the allergen-triggered sequential ϵCSR. After binding to FcϵRI expressed on mucosal effector cells, sIgE traffics to the blood stream to sensitize circulating basophils (35). After saturating FcϵRI receptors on peripheral basophils, sIgE binds to resident mast cells in peripheral tissues, including the skin (49). Only after the saturation of all receptors in the organism, sIgE can be found free in serum and other biological fluids of AR and AA patients (50). The extremely high affinity of IgE (K_d_:10^−10^) for its cognate receptor (FcϵRI) determines its binding sequence to FcϵRI+ cells in the different tissues (51).

**Figure 1:**
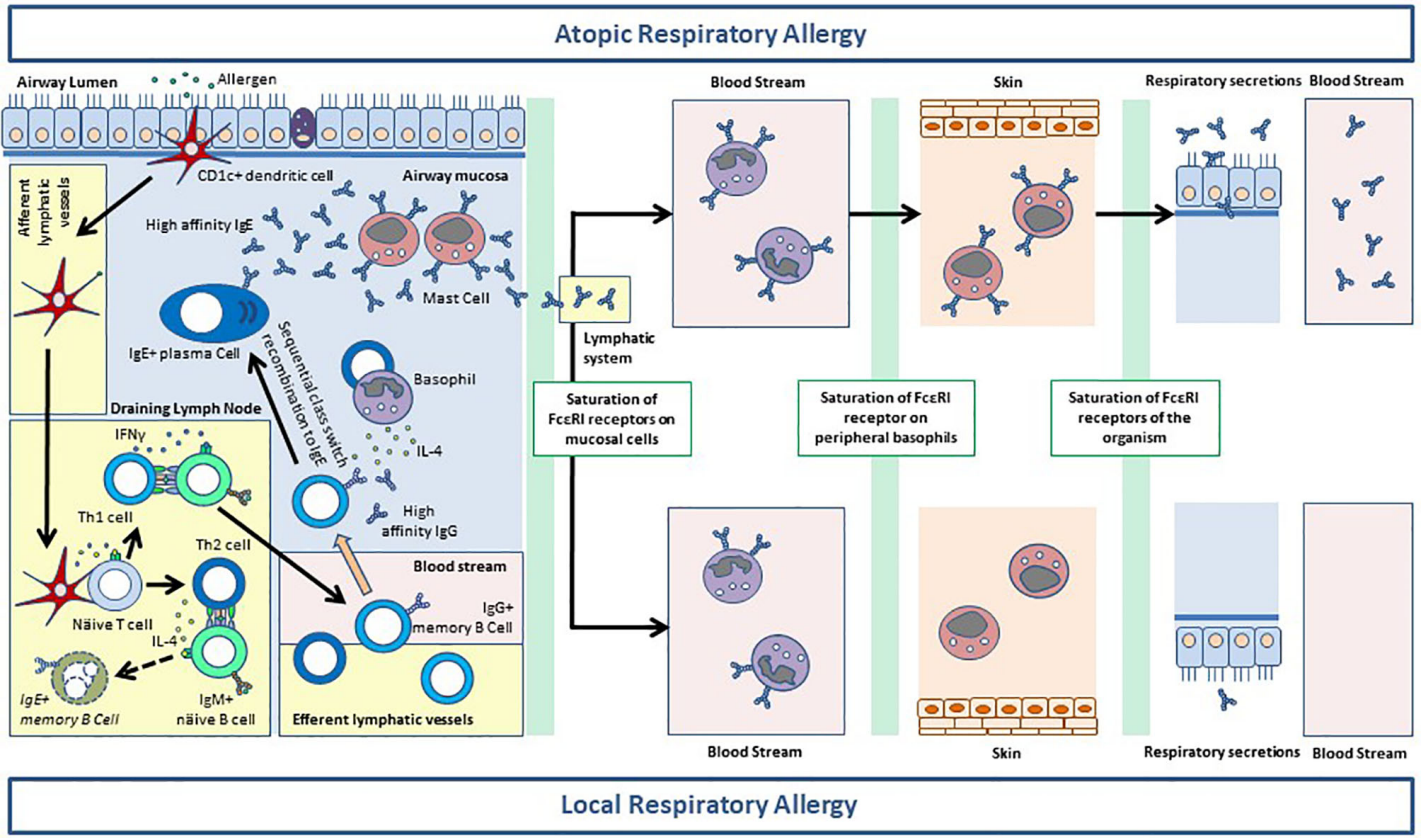
Synthesis of allergen-specific (s)IgE in the airway mucosa of atopic respiratory allergy (ARA) and local respiratory allergy (LRA) patients. CD1c+ myeloid dendritic cells take up the allergen and travel to the secondary lymphoid tissues. Here, dendritic cells activate allergen-specific naïve CD4+ T lymphocytes to give rise to Th1 and Th2 cells. Th1 cells communicate with allergen-specific IgM+ naïve B cells and promote their class switch recombination (CSR) to IgG, and their somatic hypermutation, which ultimately generates IgG+ memory B cells. The immunoglobulins produced by IgG+ memory B cells show a high affinity for the allergen. Allergen-specific Th1 and IgG+ B cells exit the lymphoid tissue and gain access to the blood stream. Th2 cells in the germinal centers also communicate with IgM+ naïve B cells to promote their CSR to IgE. Nevertheless, IgE+ B cells cannot undergo somatic hypermutation in an efficient manner, and they die by apoptosis before exiting the lymphoid tissue. On the other hand, allergen-specific Th2 cells reach the blood stream and gain access to the airway mucosa, together with Th1 cells (not shown) and IgG+ memory B cells. Upon allergen re-exposure (not shown), IgG+ memory B cells undergo sequential CSR to IgE if they are stimulated by IL-4 provided by Th2 cells or basophils. This process generates IgE+ plasma cells releasing high amounts of high-affinity sIgE. Mucosal sIgE binds to FcϵRI expressed on resident mast cells and sensitize them for activation. After saturating FcϵRI receptors in the mucosa, sIgE traffics through the lymphoid vessels to the blood stream to bind to FcϵRI on circulating basophils. In ARA subjects, there is enough sIgE to saturate the receptor system of blood basophils and sIgE binds subsequently to FcϵRI on the surface of mast cells at peripheral tissues, like the skin. After saturating the FcϵRI receptor system of the whole organism, sIgE is found free in serum and in the airway secretions of ARA individuals. In patients with LRA, the sIgE synthetized at the mucosal level is sufficient to saturate FcϵRI on mucosal resident mast cells, and in >50% of cases is also enough to sensitize peripheral basophils. Nevertheless, LRA patients do not have sufficient sIgE to saturate FcϵRI on peripheral basophils, and thus sIgE is not found on skin mast cells or serum. Most patients with LRA do not have sIgE in respiratory secretions either, although low levels are sometimes detected. This phenomenon probably corresponds to a small sIgE leakage through the epithelium, before the antibody exits the mucosa *via* the lymphatic system.

Several studies show that non-atopic eosinophilic asthmatics display markers of ϵCSR and IgE+ and FcϵRI+ cells in the bronchial mucosa (47, 48, 52). Similarly, sIgE+ cells have been found in the nasal mucosa of non-atopic rhinitis patients (53). However, in these works, the presence of mucosal sIgE was not correlated with the airway response to the allergen exposure. Another work found sIgE in the sputum of 26 out of 27 non-atopic asthmatics, but the patients displayed negative BAC results (54). Nevertheless, sputum sIgE from non-atopic asthmatics from the same study was able to activate peripheral basophils *in vitro*. Given the mechanistic diversity of non-atopic rhinitis and asthma phenotypes, it seems logical to focus the measurement of mucosal sIgE on those patients displaying positive NAC and BAC results. In this regard, sIgE is found in the nasal secretions of 20–40% of LAR patients (55–57). Nevertheless, when detected, nasal sIgE is consistently very low. Moreover, the pooled evaluation of LAR patients showed that nasal sIgE significantly increased during the 24 hours following a positive NAC, although not every individual tested positive in at least one determination (55, 56). Even though methodological aspects might partially explain this low detection yield, it cannot be dismissed that sIgE is not present in the airway secretions of most subjects with LRA. Importantly, serum sIgE is not detectable in LRA patients, and airway secretions are ultimately connected with blood through the lymphoid system. Conversely, a work utilizing Merocel^©^ sponges (a device growing inside the nostril to adapt to the anatomy, and scratching many mucosal cells upon removal) showed that >90% of LAR individuals have nasal sIgE (58). In our work defining the LAA phenotype, sIgE was not found in the sputum of any subject testing positive to the BAC (either AA or LAA patients) neither at baseline nor after the challenge (10). On the other hand, 50-66% of LAR and DAR patients have sIgE bound to the membrane of peripheral basophils (11, 59–61). This finding suggests that most LRA patients have sufficient sIgE to sensitize both airway FcϵRI+ cells and circulating basophils. In summary, although the occurrence of ϵCSR in the airway mucosa of LAR, LAA or DAR patients has not been demonstrated yet, available data suggest that the immune mechanisms of LRA and atopic respiratory allergy have many similarities (**Figure 1**).

## Diagnosis of Local Respiratory Allergy

The NAC and BAC are the gold-standard for LRA diagnosis (7). The NAC displays high safety and reproducibility (62) and counts with a validated methodology (63) and positivity cutoff points (64). Nevertheless, although highly informative, the BAC and NAC require a long stay of the patient at the hospital.

**Figure 2:**
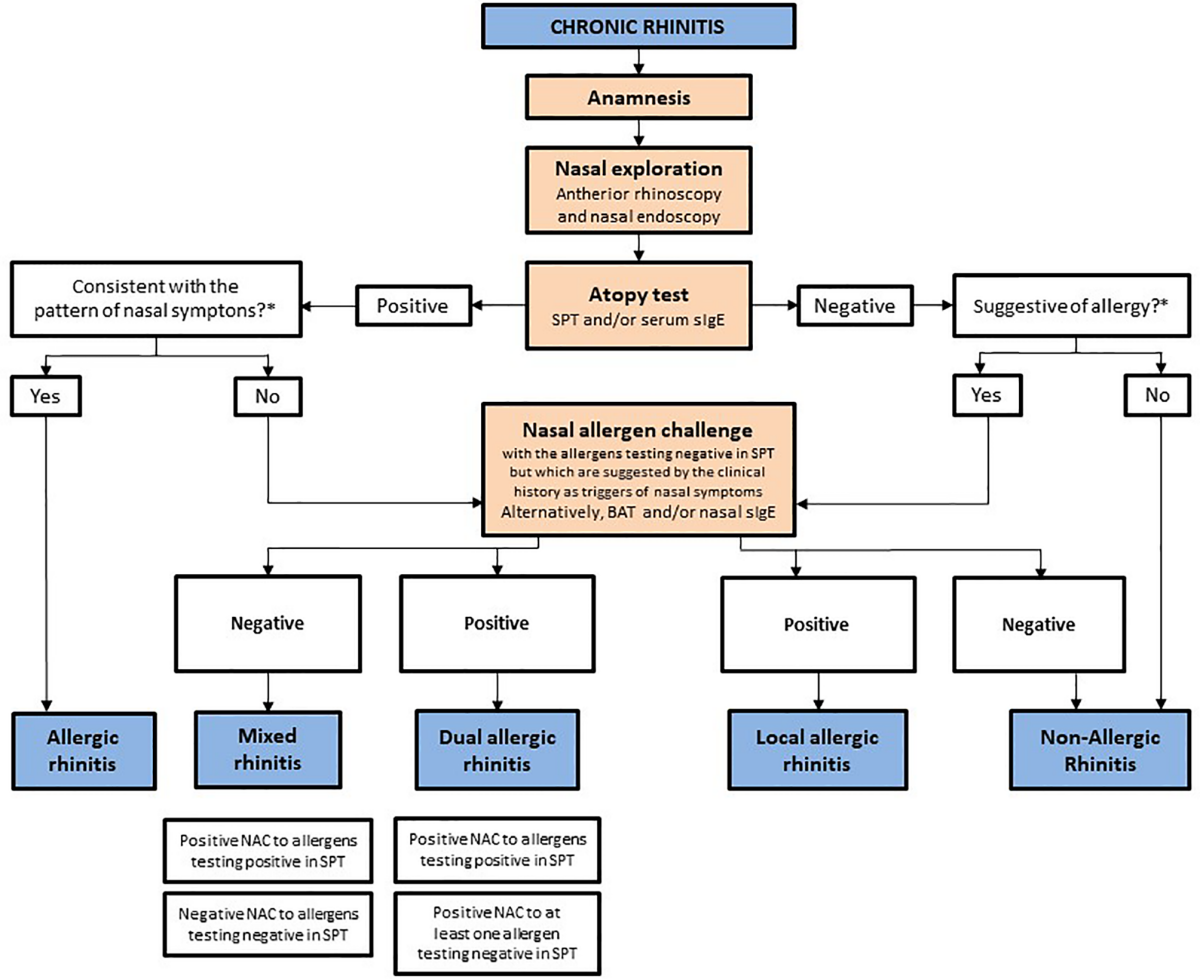
Diagnostic algorithm of chronic rhinitis phenotypes. BAT, basophil activation test; NAC, nasal allergen challenge; sIgE, allergen-specific IgE; SPT, skin prick test. *The interrogation about the seasonality and severity variations of nasal symptoms, and about the specific (vegetation, furry animals, dust, etc.) and unspecific triggers of rhinitis (rapid changes in temperature or humidity, irritant smells, etc.) are useful means to investigate the likelihood of an allergic etiology for the disease or to evaluate whether positive skin prick test results are consistent with the symptoms pattern.

In LAR patients, the quantification of sIgE in the nasal lavage is associated with low sensitivity and high specificity (100%) (55–57). Although other techniques (Merocel^©^ sponges or mucosal brushing or scraping) might improve the diagnostic performance of nasal sIgE quantification (58), the collection of these samples poses tolerability problems. On the other hand, the sensitivity of the basophil activation test (BAT) for LAR and DAR diagnosis is >50%, whereas the specificity is close to 100% (11, 59–61). Unlike mucosal sIgE measurement, the BAT counts on a validated methodology and positivity cutoff points. Moreover, the BAT informs about both the presence and functionality of sIgE (65), and is a patient-friendly technique not requiring a previous NAC to obtain maximal sensitivity (11). Nevertheless, further research is needed before the BAT can be recommended for routine LRA diagnosis. An algorithm for LAR and DAR identification among chronic rhinitis patients is shown in **Figure 2**.

## Allergen Immunotherapy for Local Respiratory Allergy

Allergen immunotherapy (AIT) is the only etiological intervention for atopic respiratory allergy. In AR and AA patients, AIT is not only able to decrease the symptoms and the use of rescue medication, but also displays a sustained effect after therapy discontinuation (66, 67). Therefore, AIT prevents the aggravation of the disease and the onset of asthma in AR patients (66, 68). Published studies indicate that during the 1^st^ year of treatment the clinical effect of AIT is achieved through the desensitization of mast cells and basophils (69). From the 2^nd^ year of treatment, AIT induces modifications in specific T and B cell clones mediating the predominance of regulatory T cells and IgG_4_+ B cells over Th2 cells and IgE+ B cells (69). These immune-modulation needs 3 years of continuous therapy (70) to get epigenetically imprinted (71), and prevails as the mechanism driving the clinical effect after therapy discontinuation (72).

The similarities between atopic respiratory allergy and LRA prompted our group to investigate the effect of AIT in LAR individuals. In 2011, we published an observational study including 20 patients with seasonal LAR due to grass pollen (73). Ten patients received pre-seasonal grass pollen-subcutaneous AIT (grass-SCIT) during 6 months, and they were also allowed to use symptomatic medication as needed. The control group was composed of 10 individuals who only took rescue medication on demand. Grass-SCIT induced a significant reduction in symptoms and medication scores, and in rhinitis severity (100% and 57% reduction of severe and moderate cases, respectively). Moreover, there was an increase in the amount of allergen tolerated in the NAC in the SCIT-treated group, with 30% of individuals testing negative to the NAC at the end of the study. Thereafter, we conducted two randomized double-blind-placebo-controlled clinical trials (RDBPCCT). The first one included 36 patients with perennial LAR due to HDM who were randomized to receive HDM-SCIT or placebo during 2 years (74). From the sixth month on, the active group showed significantly lower combined symptom and medication score (CSMS), and more medication free days. Furthermore, the nasal tolerance to the allergen was significantly higher in the active group from the sixth month, and, at the end of the trial, the actively treated individuals tolerated a concentration of HDM >3 times higher than the patients who had received placebo. The second RDBPCCT included 56 individuals with seasonal LAR due to grass pollen and had a length of 2 years (75). During the first year, patients were randomized to receive either 6 months of pre-seasonal grass-SCIT (group A) or placebo (group B), followed by 6 months of wash-up period for both study groups. The first pollen season (PS1) occurred during the wash-up period. During the second year, which included the second pollen season (PS2), both groups received 12 months of perennial grass-SCIT. The study demonstrated a significantly lower CSMS and higher frequency of medication free days in group A as compared to B during PS1. Conversely, during PS2 both groups displayed a significantly lower CSMS and more medication free days as compared to PS1 (intra-group comparison), with no significant differences between them (inter-group comparison). Moreover, for the first time, this RDBPCCT demonstrated the ability of SCIT to improve the quality of life and to decrease the conjunctival symptoms of LAR patients. At the end of the trial, the nasal tolerance to grass pollen had increased >50 times in 83% of study individuals, and 56% of them tested negative to the NAC. Interestingly, two additional RDBPCCT conducted in Poland analyzed the effect of birch-SCIT in adults with seasonal LAR and also concluded that the treatment was able to control nasal symptoms and to improve patients’ quality of life (15, 76). One of this works also showed that SCIT increases the bronchial tolerance to the allergen and reduces airway hyper-responsiveness after allergen exposure in LAA patients (15). Importantly, all four RDBPCCT show a very favorable safety profile for AIT in LAR patients (15, 74–76).

Regarding the immunological mechanisms driving the clinical effect, the four RDBPCCT showed that SCIT-treated LAR patients experience a significant increase of serum sIgG_4_ from the 6^th^ month of therapy (15, 74–76). Moreover, one of the Polish trials also demonstrates that birch-SCIT blunts the seasonal peak of nasal sIgE from the first year of treatment (76). Although published data indicates many similarities on the effect of AIT between atopic respiratory allergy and LRA patients, the long-term effect of the treatment needs to be investigated before it can be recommended for LAR, DAR and LAA patients in the daily practice.

## Conclusion

The description of LAR phenotype >10 years ago challenged the concept of atopy as the only driver of allergy. Nowadays, it is apparent that atopy and allergy represent two different phenomena requiring distinct identification methods. Current evidence indicates that local allergic mechanisms do not only affect LAR patients, but are also involved in other rhinitis and asthma phenotypes. Therefore, there is a need to update the classifications of airway diseases in order to progress towards a more endotype-based division. This advance will favor the recognition of LRA patients in the clinic, and will subsequently allow for early interventions with disease-modifying potential.

## Author Contributions

IE-G and CR conceptualized the article. AT-M, MS, FP, and AA critically reviewed the literature and drafted the manuscript. MT, CR, and IE-G reviewed the manuscript and supervised the work of the other authors. All authors contributed to the article and approved the submitted version.

## Funding

This work was supported by the Instituto de Salud Carlos III of the Spanish Ministry of Science and Competitiveness (grants co-funded by the European Regional Development Fund) through the research contracts “Rio Hortega” for AT-M (CM20/00160) and “Juan Rodes” for IE-G (JR19/00029), the research project PI17/01410 and PI20/01715 and the program of Redes Temáticas de Investigación Colaborativa en Salud (RETICS): Asma, Reacciones Adversas y Alérgicas-ARADyAL (RD16/0006/0001). FP holds a “Stop Fuga de Cerebros” grant from Roche (SFC-0002-2020). This work was also supported by the Andalusian Regional Ministry of Health through the research project PC‐0098‐2017 and PI-0176-2018.

## Conflict of Interest

The authors declare that the research was conducted in the absence of any commercial or financial relationships that could be construed as a potential conflict of interest.

## Abbreviations

AA, allergic asthma; AIT, allergen immunotherapy; AR, allergic rhinitis; BAC, bronchial allergen challenge; BAT, basophil activation test; CSMS, combined symptoms and medication score; CSR, class switch recombination; DAR, dual allergic rhinitis; ϵCSR, class switch recombination to IgE; ECP, eosinophil cationic protein; HDM, house dust mites; LAA, local allergic asthma; LAR, local allergic rhinitis; LRA, local respiratory allergy; MR, mixed rhinitis; NAC, nasal allergen challenge; NAR, non-allergic rhinitis; RDBPCCT, randomized double blind placebo-controlled clinical trial; sIgE, allergen-specific IgE; sIgG_4_, allergen-specific IgG_4;_ SPT, skin prick test.
